# Effects of improved information and volunteer support on segregation of solid waste at the household level in urban settings in Madhya Pradesh, India (I-MISS): protocol of a cluster randomized controlled trial

**DOI:** 10.1186/s12889-021-10693-0

**Published:** 2021-04-09

**Authors:** Madhanraj Kalyanasundaram, Yogesh Sabde, Kristi Sidney Annerstedt, Surya Singh, Krushna Chandra Sahoo, Vivek Parashar, Manju Purohit, Ashish Pathak, Cecilia Stålsby Lundborg, Kamran Rousta, Kim Bolton, Salla Atkins, Vishal Diwan

**Affiliations:** 1 Division of Environmental Health and Epidemiology, ICMR – National Institute for Research in Environmental Health, Bhopal, 462 030 India; 2 Department of Global Public Health, Karolinska Institutet, SE-171 77 Stockholm, Sweden; 3 Division of Environmental Monitoring and Exposure Assessment (Water & Soil), ICMR – National Institute for Research in Environmental Health, Bhopal, 462 030 India; 4 ICMR- Regional Medical Research Centre, Bhubaneshwar, 751023 India; 5 Department of Public Health and Environment, RD Gardi Medical College, Ujjain, 456006 India; 6 Department of Pathology, RD Gardi Medical College, Ujjain, 456006 India; 7 Department of Paediatrics, RD Gardi Medical College, Ujjain, 456006 India; 8 Department of Women and Children’s Health, International Maternal and Child Health Unit, Uppsala University, SE-751 85 Uppsala, Sweden; 9 Department of Resource Recovery and Building Technology, University of Boras, 50190 Boras, Sweden; 10 New Social Research and Global Health and Development, Faculty of Social Sciences, Tampere University, 330 14 Tampere, FI Finland

**Keywords:** Urban, Household waste segregation, Randomised controlled trials, Solid waste management, Protocol, India

## Abstract

**Background:**

Segregation of household waste at the source is an effective and sustainable strategy for management of municipal waste. However, household segregation levels remain insufficient as waste management approaches are mostly top down and lack local support. The realisation and recognition of effective, improved and adequate waste management may be one of the vital drivers for attaining environmental protection and improved health and well-being. The presence of a local level motivator may promote household waste segregation and ultimately pro-environmental behaviour. The present cluster randomized control trial aims to understand if volunteer based information on waste segregation (I-MISS) can effectively promote increased waste segregation practices at the household level when compared with existing routine waste segregation information in an urban Indian setting.

**Methods:**

This paper describes the protocol of an 18 month two-group parallel,cluster randomised controlled trialin the urban setting of Ujjain, Madhya Pradesh, India. Randomization will be conducted at ward level, which is the last administrative unit of the municipality. The study will recruit 425 households in intervention and control groups. Assessments will be performed at baseline (0 months), midline (6 months), end line (12 months) and post intervention (18 months). The primary outcome will be the comparison of change in proportion of households practicing waste segregation and change in proportion of mis-sorted waste across the study period between the intervention and control groups as assessed by pick analysis. Intention to treat analysis will be conducted. Written informed consent will be obtained from all participants.

**Discussion:**

The present study is designed to study whether an external motivator, a volunteer selected from the participating community and empowered with adequate training, could disseminate waste segregation information to their community, thus promoting household waste segregation and ultimately pro-environmental behaviour. The study envisages that the volunteers could link waste management service providers and the community, give a local perspective to waste management, and help to change community habits through information, constant communication and feedback.

**Trial registration:**

The study is registered prospectively with Indian Council of Medical Research- Clinical Trial Registry of India (CTRI/2020/03/024278).

## Background

Globally, solid waste management is a major environmental issue. As of 2016, annual global Municipal Solid Waste (MSW) generation levels are 2.01 billion metric tonnes (MTs), with the per capita waste generation of 740 g per day. Owing to rapid urbanization and population growth, waste generation is expected to increase by 70% from 2016 levels to 3.4 billion MTs per year in 2050 [1, 2]. Proper segregation and disposal of generated waste is one of the important challenges being faced globally for attaining environmental protection and improved health and well-being [3–5]. Low- and middle-income countries (LMICs) specifically face continuous challenges in providing sustainable waste management services to their population due to the lack of organisation, financial resources, and the systems’ complexity [4, 6–8]. The importance of solid waste management has been recognized and embedded in all the Sustainable Development Goals (SDGs) either directly or indirectly. Hence, progress in solid waste management is important to deliver almost all of the SDGs [9].

India, the second populous country in the world, generates 147.6 million metric tonnessolid waste per day. The per capita waste generation ranges from 170 g to 620 g per day with an annual increase of 1.3% [2]. Recently the Government of India developed strategic plans, updated regulatory frameworks for solid waste management and started many initiatives to achieve sustainable waste management systems for Indian cities [10]. Despite these efforts, the progress in solid waste management in India has been slow. One of the factors responsible for the slow progressis the ‘top-down approach’ [10, 11]. Constitutionally, solid waste management comes under the purview of Urban Local Bodies (ULBs) [12]. However, in reality community participation in the process of solid waste management is often neglected [10, 12]. Further, there is limited environmental awareness among the public resulting in public attitudes creating barriers to improving waste management [13].

Studies have shown that empowering the community by providing appropriate knowledge and motivation, with an enabling environment and involving the community in waste management processes result in improved waste management practices at the household level, for example increased waste segregation and recycling behaviour [14, 15]. However, studies have also identified that sustaining the changed behaviour is the biggest challenge [16]. An external motivator and/or a local volunteer could promote sustainable change in household waste segregation practices and ultimately pro-environmental behaviour [17–20]. The volunteers could act as a link between the waste management services and the community, give a local perspective to waste management, and help change community habits through information, and constant communication and feedback, thus addressing many of the identified barriers. Volunteer-based interventions can also help in identifying other, contextually appropriate, ways of waste management [21, 22]. To our knowledge there are no such interventions reported in Indian context.

Therefore we propose the study to measure the impact of volunteer based one-on-one delivery of improved waste segregation information at household level. As the intervention is at household level and targeting behaviour change at household level as an outcome, we chose to adopt a cluster-randomized controlled trial (c-RCT) design to reduce the risk of potential contamination.

This paper describes the study protocol of an 18 month two-group parallel, c-RCT that aims to examine the effects of improved information and volunteer support on waste segregation at the household level in urban settings of Ujjain in Madhya Pradesh, Central India.

## Methods

### Hypothesis

We hypothesize that in urban settings of Central India, a volunteer based delivery of improved information on waste segregation (I-MISS) fortnightly for 12 months at household level in addition to the routine/ general information on waste segregation given by the municipality, will improve household waste segregation practices when compared to the households receiving only routine/general information on waste segregation given by the municipality.

### Objective

To examine the effects of improved information and volunteer support on waste segregation practices at the household level in urban settings of Ujjain in Madhya Pradesh, India.

### Study design

**Fig. 1:**
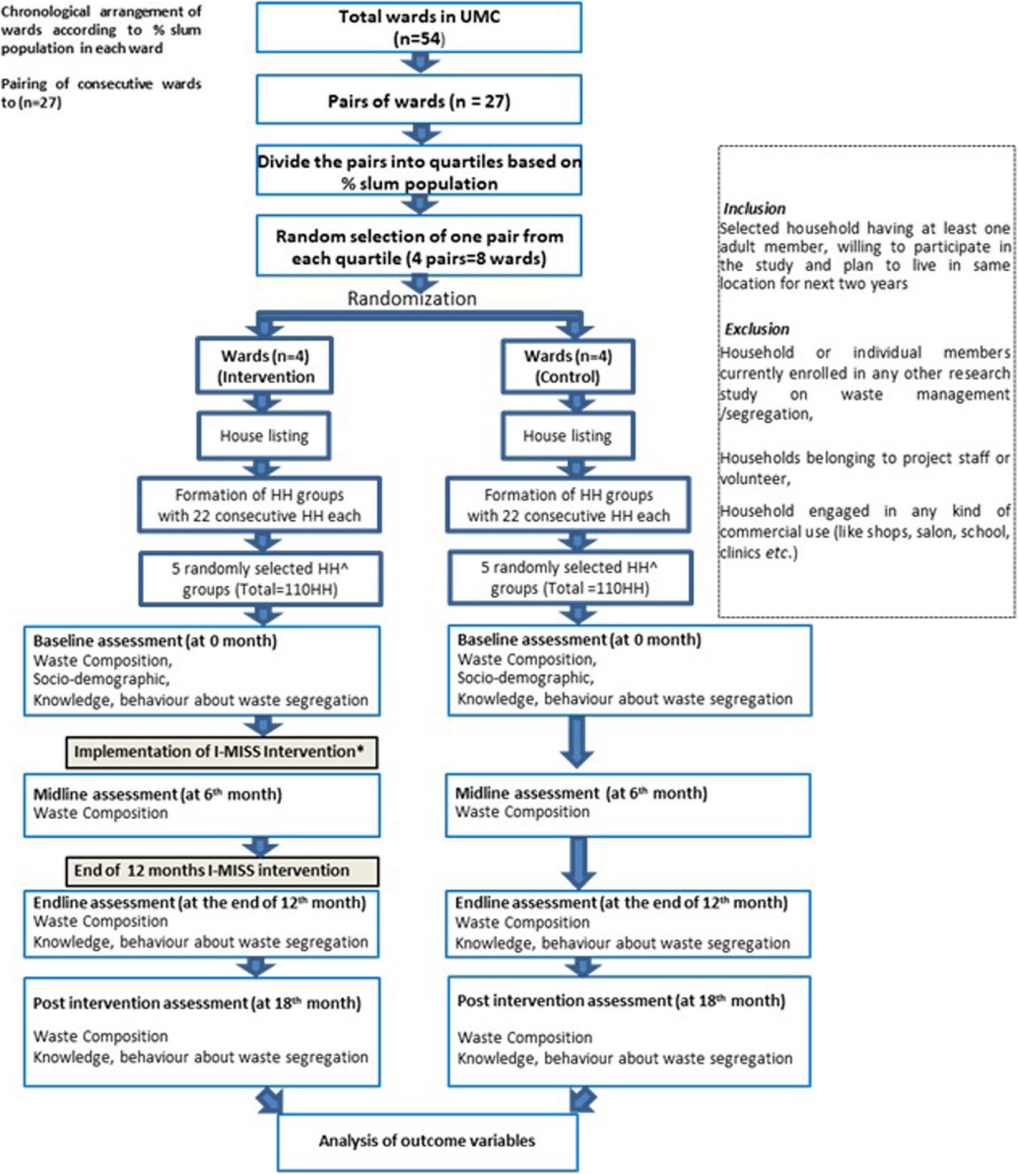
Design and participants flow chart according to SPIRIT 2013 guidlines (Standard protocol items: Recommendations for International Trials)

This is an 18 month two-group parallel, c-RCT. The trial will be conducted following the Consolidated Standards of Reporting Trials (CONSORT)- extension for c-RCT guidelines [23]. The protocol is prepared in agreement with the standard protocol Items from the recommendations for interventional trials (SPIRIT) guidelines [24]. In our study, we will randomize at ward level i.e., wards will be our cluster randomization unit. Wards are the last administrative unit of municipality. The wards will be stratified into four stratum based on the percentage of slum population within a ward. From each stratum, wards will be randomized into intervention and control arms with 1:1 allocation ratio. The study design and trial flow are depicted in Fig. 1.

### Study setting

This study will be conducted within the area of the Ujjain Municipal Corporation in Madhya Pradesh, Central India. Ujjain city is a medium sized city in India with a municipal area of 93 sq. km. The population is 0.56 million and the slum population is around 23.32% of the total population [25, 26]. The Ujjain Municipal Corporation area is comprised of six zones and 54 administrative wards [25]. Nearly 225 MTs of total municipal solid waste is generated in Ujjain City daily [27].

### Study Population

Households located within the administrative boundary of Ujjain Municipal Corporation will be our study population.

#### Inclusion criteria

We will include households with at least one member above 18 years of age located within the administrative boundary of Ujjain Municipal Corporation with domiciliary stability of about 2 years.

#### Exclusion criteria

- Households or individual adult members currently enrolled in any other research study on waste management /segregation.
- Households of volunteer and project staff
- Household whose waste mixes with waste generated from commercial use (Shops, markets, salon, school, clinics etc.,) and industrial use.

#### Definition of household and slum household

We will follow the definition of household given by Census of India i.e., A ‘household’ is usually a group of persons who normally live together and take their meals from a common kitchen unless the exigencies of work prevent any of them from doing so. There may be one member households, two member households or multi-member households [28].

We will follow the slum definition given by Census of India [28, 29] in this study. According to them, slums are categorized and defined as one of the following three types:

I. **“Notified slums** (All notified areas in a town or city notified as ‘Slum’ by State, Union Territory (UT) Administration or Local Government under any Act including a ‘Slum Act’)”
II. **“Recognized slums** (All areas recognised as ‘Slum’ by State, UT Administration or Local Government, Housing and Slum Boards-not been formally notified as slum under any act)”
III. **“Identified slums** (A compact area of at least 300 population or about 60-70 households of poorly built congested tenements, in unhygienic environment usually with inadequate infrastructure and lacking in proper sanitary and drinking water facilities- identified by the census of India)”

Any household located within the above mentioned areas will be considered as slum households.

##### Sample size

The sample size will be based on the differences in proportion, assuming that 50% households segregate waste in both groups. As we could not find previous literature quantifying the effect of similar kind of intervention in Indian setting, we assumed that in our study the intervention will increase the waste segregation practice in the intervention group by 20% compared to the control group. Given a 5% significance level and 80% power, then the minimum sample size is *n* = 184 per group. Assuming a design effect of 2 to adjust for the clustering effect, with 10% non-response rate and 20% loss to follow-up, the final sample size for each arm will be 425 households (total 850 households).

We have fixed the cluster number as eight. As the required sample size is 850 households, we will recruit 850 households from 8 wards i.e., around 110 households (cluster size) will be recruited from each ward.

### Sampling strategy

The Ujjain Municipal Corporation is divided into 54 wards. Wards are the last administrative unit of the municipality. Wards will be our randomization unit (cluster).

All the wards in Ujjain Municipal Corporation (*n* = 54) will be chronologically arranged according to percentage of slum population in each ward. The consecutive wards will be arranged into pairs (*n* = 27 ward pairs). These pairs will be divided into quartiles based on the percentage of slum population. One ward pair will be selected by simple random sampling from each quartile. Thus, a total of four ward pairs (eight wards) will be selected. The lottery method (simple randomization) will be used to randomly allocate wards in each pair into intervention and control groups.

A survey team will conduct a preliminary door to door survey to list the total number of households in the selected eight wards. They will gather information of slum and non-slum households. Thus, at the end of this process, we will have a sampling frame (line listing of households classified as slum and non-slum areas).

After the household listing, each ward (cluster) will be divided into groups of 22 consecutive households. To meet the required sample size, we will identify at least five groups of 22 households from each ward (5X22 = 110 households). In total, 440 households will be recruited for the intervention group and another 440 for the control group.

### Intervention details

#### Intervention group

The intervention will be at household level. Households selected for the intervention arm will be visited fortnightly by a volunteer who will give improved waste segregation information. This intervention will be in addition to the routine /general information on waste segregation that is given by the municipality.

The volunteer will be an adult individual recruited from the same cluster (ward) as their households (permanent resident of the locality) able to read and write the local language, with interest and willingness to spend time to improve waste management in their residential area. The intervention will be delivered primarily through one to one interactive sessions. The interaction will be in the local language supported by flip cards, pictures, and visuals. The volunteer will visit fortnightly for 12 months, meaning that a volunteer will visit a household at least 24 times during the intervention period. The total duration of each interaction session will be about 15–30 min. At least primary and/or secondary members of households responsible for waste disposal identified in the baseline survey by self-reported method must be available in these interactive sessions. However, all other available and interested adult members will be included in the sessions. Attendance of presenting members and any other information shared by household members will be systematically recorded by the volunteer in a logbook. The broad topics to be covered in the improved information include types of waste, correct waste segregation methods, residents’ role and responsibilities, waste management and its effects on health and wellbeing. Standard operating procedure (SOPs) will be prepared, so that the intervention package will be standard in all intervention households to avoid the risk of intervention variability and to increase intervention fidelity. One volunteer will be allocated to approximately 22 households. The volunteer will be paid small monetary incentive for their role in the project. At least two induction training programmes will be organized for volunteers to train the volunteers in all aspects of intervention content and SOPs to ensure that the intervention is delivered uniformly across households. They will be also trained in communication skills. Two refresher trainings are planned during the intervention period (4th month and 8th month).

### Control group

The households in the control arm will receive the waste segregation information dissemination campaign conducted by Ujjain Municipal Corporation, which is routine and same across the city.

In addition, all the participating households (Intervention and control arm) will be provided with a pair of dustbins to enable waste segregation practice.

### Intervention (I-MISS) content development

The content will be developed based on extensive literature review and expert consultations. Experts from the field of public health, medical doctor, epidemiologist, social scientist and waste management researchers, providers from municipality involved in solid waste management and NGOs will be included in expert consultations. Textual and content analysis will be conducted for the qualitative data. Themes and sub themes emerged will be pinned under the framework suggested in the motivation-opportunity-ability-behavior (MOAB) model [30–33]. The model explains that beliefs, evaluation of outcomes and social norms bring in the attitude towards the targeted pro environmental behaviour (In this case waste segregation), into what is collectively called the Motivation box. The intention for the target behaviour is the immediate antecedent for the behaviour/action in this model. Elements like ability and opportunity, will be considered as moderators to convert intention into action. The model also establishes a return connection between behaviour and belief directly or via ability which shows that when behaviour becomes a habit, the belief and evaluation of outcomes of thisbehaviour can be changed towards stronger attitudes [31–33].

The intervention content will include comprehensive information about types of household waste, correct waste segregation procedure, residents’ role and responsibilities, waste segregation and management and its impact on health, environment and wellbeing informed by the above consultation. The content will be organized so that volunteers will be able to deliver the I-MISS intervention in approximately 15–30 min per household session. The face and content validity of the developed intervention will be assessed. The intervention will be piloted in settings similar to the intervention areaswithin the Ujjain Municipal Corporation.

### Blinding

As the intervention consists of training that targets a change in behaviour, blinding of the study participants and investigators is not possible. However, the study team responsible for conducting outcome assessments will be blinded in terms of the households’ allotted groups and the participants will be requested to not discuss their allocation with the research team responsible for conducting follow-up assessments in order to minimize bias.

### Allocation concealment

The sampling procedure and randomization of wards will be performed by an external statistician. The list of selected wards will be communicated to the study investigators to initiate line listing, recruitment and baseline survey. However, at this stage the allocation of wards to intervention/control group will be concealed (by sealed envelope) by the external statistician. Allocation will be revealed after the baseline survey and before the initiation of intervention.

### Study measures

#### Primary outcome measures

- Comparison of change in proportion of households practising waste segregation at different time points in the study when compared to baseline, between the intervention and control groups (Difference in differences between the groups). This is a categorical self-reported variable assessed at household level which will be captured by the waste segregation knowledge and behaviour questionnaire. The variable will reflect whether or not the household is segregating solid waste at household level, but will not reflect the accuracy of segregation.
- Comparison of change in proportion of mis-sorted waste at different time points in the study when compared to baseline between the intervention and control groups (Difference in differences between the groups). This variable will be objectively assessed at group level across intervention and control group by standard waste composition analysis (pick analysis). This variable will reflect the accuracy of waste segregation practices at group level.

#### Secondary outcome measures

- Change in quantity (in mass) and waste composition at different time points in the study when compared with the baseline between the intervention and control groups as assessed by pick analysis
- Comparison of change in knowledge and practices about solid waste management at household level at different study time points when compared to the baseline between the intervention and control group (assessed by *Waste segregation knowledge, behaviour questionnaire)*

### Study tools

- *Pick analysis (waste composition assessment): *Pick analysis is a method to characterize the content of a waste stream. This analysis aims to estimate the waste generation per capita per time and to analyse waste segregation behaviour where waste fractions were sorted correctly based on the recycling schemes. This method can measure the actual waste sorting behaviour at household/community level. The method was developed and validated by Dahlén and Lagerkvist [34] and is a recommended method by Swedish Waste Management Association [35](2013). Rousta et al. (2016) have implemented this method to create RBT procedure (Recycling behaviour Transition) to design and assess the interventions of increasing the recycling rate of a community in a medium-sized city [31]. A pre- designed proforma to capture the quantity and composition of the waste generated at group level will be used.
- *Waste segregation knowledge, behaviour questionnaire***:** Based on the literature [11, 13, 22, 36–41] and experts’ consultation, the research team has developed an extensive questionnaire to assess the awareness, attitude and behaviour related to household level waste segregation. This questionnaire will be used to assess the belief, social norms, knowledge, ability, motivation factors, opportunity and practices about waste segregation study household members. One section of the questionnaire will capture socio-demographic details of the study households.
- *Intervention log-book (Volunteer log book):* A pre-designed log book will be used by the volunteers to monitor the attendance of household members present during intervention and any other information shared by household members.

### Testing and validation of study tools

All assessment questionnaires will be validated using face and content validation method for comprehensibility, simplicity, relevance and cultural acceptability. Pretesting of the study tools will be done with randomly selected households (*n* = 30–50) not part of the trial to assess the overall feasibility of the proposed intervention, improve the intervention implementation, and inform the design of a tailored intervention.

### Assessments (Table 1)

Assessment will be conducted at four time points (baseline, midline, end line and post intervention) across the study period.

- *Baseline: *At baseline, all the households (intervention and control group households) will be assessed for the following:
  - ○ Waste composition assessment through pick analysis, which is a reliable and validated method to assess the composition of the waste generated at group level.
  - ○ General socio-demographic details and waste segregation knowledge and behaviour.
  Findings of waste composition at baseline will be shared with respective households and two dustbins will be provided to each household (intervention and control) with relevant information regarding waste segregation.
- *Midline: *At the halfway point of the intervention (6th month), waste composition analysis of all the households (intervention and control group households) will be conducted using the pick analysis method.
- *End line:* At the 12^th ^intervention month waste composition, waste segregation knowledge and behaviour will be assessed again using the same methods as in the baseline assessment.
- *Post Intervention ***:** Waste composition, waste segregation knowledge and behaviour will be assessed finally at the 18 month mark (6 months after intervention completion). Feedback about the intervention will be collected from participants at this assessment.

**Table 1:** Assessment plan

Time Point	Baseline (M-0)		Intervention period (12 Months)				Post Intervention (M-18)	
			Midline (M-6)		End line (M-12)			
Study tools	Intervention	Control	Intervention	Control	Intervention	Control	Intervention	Control
Socio-demographic	X	X	–	–	–	–	–	–
Waste composition analysis	X	X	X	X	X	X	X	X
Knowledge and behaviour, about waste segregation	X	X	–	–	X	X	X	X
**Fortnight visit for I-MISS Intervention**								

### Intervention fidelity

Standard operating procedure (SOPs) will be prepared, to ensure a standard intervention package in all intervention households. Adequate induction and refresher trainings for volunteers in all aspects of intervention content and for SOPs will be conducted. Participating households in the intervention arm will be contacted telephonically monthly by a separate team (not involved in outcome assessments) to get feedback about the intervention delivery by the volunteers and to ensure visits have been conducted. The investigators will conduct a monthly meeting with the volunteers to collect their feedback on the trial process and implementation*.*

### Loss to Follow-Up

Where households are not available for assessments the project team will make multiple (approximately 5) attempts to contact the head of household or primary/secondary member responsible for waste disposal or any members of the household to reschedule the missed visit. The investigator will ensure every effort to regain contact with the household before classifying a household as lost to follow up. These contact attempts will be documented in the trial record. Thus, if the team fails to make contact of the households after 5 attempts or if the household respondents revoke the consent to participate in the study will be labelled as lost to follow up.

### Ethical Considerations and approvals

The study is approved by the institutional ethics committee of ICMR-National Institute for Research in Environmental Health, Bhopal (No. NIREH/BPL/IEC/2020–21/41, dated 21st April, 2020) and R.D. Gardi Medical College, Ujjain (No.03/2020, dated March 12th 2020). The study is approved by Health Ministry Screening Committee (2020/9308). The study is prospectively registered in Clinical Trials Registry India (CTRI/2020/03/024278).

Written informed consent will be obtained from all participants. Households will be given the option to withdraw from the study at any time without justification. If the household decides to withdraw from the study, the research team may keep and continue to use any data collected before the withdrawal. However, if a household withdraws from the trial, they may request destruction of any data collected.

The objective of this trial is to assess the change in waste segregation practice using volunteer based improved information which are not anticipated to cause any adverse events or risk to participating households. However, study or follow-up assessments may create additional stress of segregating waste, but unlikely to such an extent that it will cause harm. We anticipate no or less than minimal psychological discomfort related to waste segregation activities and frequent home visits made by project staff. Each visit will be done at convenient time for the study participants. Only interested participants will be involved in the study. All ethical procedures will be followed across the study period.

Data confidentiality will be guaranteed through well-established protocols for data collection and processing. Unique identifier will be assigned to participants. The key linking the personal data with related results will be kept inside locked cabinets and shall be accessed only by the authorized staff. Paper-based forms will be filled to document epidemiological data (e.g. socio-demographics and other information). These forms will be kept under the custody of the authorized in-charge manager and subsequently entered in electronic database. Hard copies of all the data collection forms will be stored in a locked archive with restricted access. Electronic data will be stored in password-protected computers in restricted working areas accessible to project staff only. Incremental back-up files will be produced weekly. Any participant records or datasets that are transferred to other participating institute will contain the identifier only; participant names or any information which would make the participant identifiable will not be transferred. The results of proposed trial will be disseminated through community presentations, workshops, policy briefs, in meetings, in conferences and through publications in peer-reviewed journals. Any amendments in the approved proposal during implementation will be communicated to all the institutional ethics committees of participating institutes.

### Strategies to Maintain Adherence

To reduce participant attrition, an initial face to face information session among participant households will be conducted prior to the project start. The project team will inform participants about the study objectives and address queries and concerns. Participants will be contacted monthly for their feedback and also to address their queries and concerns.

### Community Advisory Board

A community advisory board (CAB) will be setup. The board will comprise of waste management professionals, community members and academicswho will hold dialogue with the study participants about the study. CAB members will also give feedback to the project team.

### Database management

The principal investigator will oversee the process of data management. REDCap will be used for database management. Participants will be given a unique participation information number (PIN) to ensure anonymity and data confidentiality. The entered data will be analysed using to appropriate statistical software. Descriptive statistics will include the mean, median, standard deviation and Interquartile range to summarize the numerical variables. Frequencies and percentages will be used for the categorical variables. Student t test will be used to compare means between the groups and chi square test will be used to compare the percentages between the groups. For skewed data, appropriate non parametric tests will be used. ANOVA repeated measures will be used to compare the means across the study periods (multiple time points) within the groups. Factorial ANOVA will be used to compare the means between the groups across the study period (multiple time points). Appropriate post hoc test (Bonferroni test) will be applied. Multi-level modelling (MLM) and Generalized Estimating Equation (GEE) will be done to adjust the clustering effect and to compare the proportions between the groups across the study period. Intention-to-treat analyses will be followed to assess interventions’ effectiveness.

### Project Coordination Committee

The Project Coordination Committee (PCC) will be formed for effective coordination, management, planning and implementation of project. The PCC will be comprised of PI and co-investigators. Additional persons may be included, where appropriate The PCC will meet face to face once in a year along with annual meeting. PCC will also be in touch during the entire life of project through telephone, emails and other online meeting platforms.

### Data Collection and Management Committee

Data Collection and Management Committee (DCMC) will be formed. The DCMC will have at least one person from each partner organization having specific responsibility for data collection and management. The DCMC will be responsible for 1) designing study tools, intervention and databases 2) development of protocol for data collection, management, entry and quality check 3) trainings of volunteer and research staff 4) protocol for data storage and documentation (4) Handle all related issues including ethical aspect 5) communication and dissemination.

### Quality control

Will be applied to each stage of data handling to ensure that all the data are reliable and have been processed correctly. The research team will perform source data review and source data verification to confirm that data entered by authorized personnel are accurate, complete, and verifiable from source documents; that the safety and rights of participants are being protected; and that the trial will be conducted in accordance with the currently approved protocol and any other trial agreementsand all applicable regulatory requirements.

## Discussion

Improved solid waste management at all levels is needed to maintain environmental balance amid rapid socio-economic development [42, 43]. Adopting proper waste segregation practices at household level could be the key for developing workable waste management systems in urban settings. Segregation at the source ensures that waste goes through different recycling and resource recovery streams, reducing waste and offering economic opportunities for households and communities [4]. Waste segregation and its proper management can be one of the important determinants of creating healthy, and sustainable communities. In the context of the SDGs, realisation and recognition of effective, improved and adequate waste management levels may be the key, vital driver for attaining environmental protection and improved health and well-being [44].

Waste management in LMICs is highly centralized and the role of community participation in waste management is limited [45]. The existing system usually adopts a legal route of collecting fees or fining for noncompliance to change peoples’ behaviour. The sustainability of behaviour change achieved by such approaches is questionable. Innovative, context-specific, economical, socially acceptable, participatory, simple, evidence-based interventions are needed for developing sustainable waste management solutions [46, 47]. The present study is designed with the concept that an external motivator, who will be the volunteer selected from the own community, empowered with adequate training, could disseminate the information to their community and may promote household waste segregation and ultimately pro-environmental behaviour. The study envisages that the volunteers could act as the link between waste management service providers and the community, give a local perspective to waste management, and help to change community habits through information, and constant communication and feedback. Previous studies have shown that volunteer based education intervention improved environmentally responsible behaviours e.g. recycling at the household level [31, 37, 40, 41, 45, 48, 49]. This technique is believed to work due to individuals being motivated by appearing consistent. This method may also help to other ways of waste management appropriate to context. Building on the results and the experience gained from this study, we could develop better, more efficient interventions to promote pro-environmental behaviour. There are studies suggest that interventions developed with an underpinning of an explicit theoretical foundation are more effective than those lacking a theoretical base [50]. In our study, the intervention content will be developed based on the integrated MOAB model which incorporates the complexity of behaviour and often used to influence waste segregation and recycling behaviour [30, 32]. Thus, we envisage that combining the concept of local volunteer as an external motivator delivering an information developed with an explicit theoretical foundation could be effective.

The objectives of the present study are well connected and relevant to the Government of India’s initiatives of Clean India Mission and development of Smart Cities that aim to develop citizen friendly and sustainable cities across India. This initiative also includes effective waste segregation and management systems in urban communities as a focus [51]. The findings of this project will add to understanding of households’ compliance and challenges of waste segregation. The results of the proposed intervention to improve waste segregation can be replicated in neighbourhood clusters and similar settings on a broader scale. The project processes and results may add social value contributing to improved health and well-being [41].

### Methodological Considerations

The project is built on the interdisciplinary strengths of the teams in the Sweden and India. Leveraging each team’s complementary intellectual contribution whilst providing opportunities for learning and capacity building across the teams is one of the strengths of the study. Adoption of behaviour change theory and multiple methods to develop the intervention is considered as one of the strengths of the study. The adoption of sound study design (c- RCT) as per CONSORT guidelines [23] and comprehensive sampling strategy with adequate sample size will lead to generate high level evidence which could be extrapolated to population level.

The study households /participants will not be blinded regarding the group they were allocated. This could exaggerate the difference in outcomes measurement between the two groups. Also, the attention given to intervention participants during home visits by volunteers, in itself may raise adherence (through the Hawthorne effect). Although volunteers will ensure that all household members should participate in fortnightly session, it is possible that some household members will not be there for these sessions. Errors may be made while measuring waste fraction during waste composition study. To minimize uncertainty in the data collection, all waste composition measurements at different time points will be conducted in presence of senior project staff with the same supporting staff. The same measuring equipment and protocol will be followed in all measurement sessions.

## Data Availability

Not applicable (Data sharing does not apply to this article which describes a study protocol and thus no datasets have been generated or analysed yet).

## Abbreviations

MSW: Municipal Solid Waste
MTs: Metric Tonnes
ANOVA: Analysis of variance
CAB: Community Advisory Board
CONSORT: Consolidated Standards of Reporting Trials
c-RCT: Cluster Randomized Controlled Trial
DCMC: Data Collection and Management Committee
GEE: Generalized Estimating Equation
LMICs: Low and Middle Income Countries
MLM: Multi-level modelling
MOAB: Motivation-Opportunity-Ability-Behavior
NGOs: Non Governmental Organization
PCC: Project Coordination Committee
PIN: Participation Information Number
RBT: Recycling Behaviour Transition
RCT: Randomized Controlled Trial
SDGs: Sustainable Development Goals
SOPs: Standard Operating Procedures
SPIRIT: Standard protocol Items from the recommendations for interventional trials
ULBs: Urban Local Bodies
UT: Union Territory
